# Reputation management promotes strategic adjustment of service quality in cleaner wrasse

**DOI:** 10.1038/s41598-017-07128-5

**Published:** 2017-08-21

**Authors:** Sandra A. Binning, Olivia Rey, Sharon Wismer, Zegni Triki, Gaétan Glauser, Marta C. Soares, Redouan Bshary

**Affiliations:** 1 Université de Neuchâtel, Institut de Biologie, Eco-Ethologie, Rue Emilie-Argand 11, 2000 Neuchâtel, Switzerland; 2 Université de Neuchâtel, Institut de Chimie, Neuchâtel Platform of Analytical Chemistry, Avenue de Bellevaux 51, 2000 Neuchâtel, Switzerland; 3 CIBIO, Centro de Investigação em Biodiversidade e Recursos Genéticos, Universidade do Porto, Vairão, Portugal

## Abstract

Adjusting one’s behaviour in response to eavesdropping bystanders is considered a sophisticated social strategy, yet the underlying mechanisms are not well studied. Cleaner wrasse, *Labroides dimidiatus*, cooperate by eating ectoparasites off “client” fishes, or cheat (i.e. bite) and eat client mucus. Image scoring by bystander clients generally causes cleaners from socially-complex (i.e. high cleaner and client abundance; high client species richness) habitats to increase levels of cooperation. However, some individuals may periodically provide tactile stimulation to small resident clients, which attract bystanders close that are bitten, a form of tactical deception. Cortisol injection can reproduce this pattern. Here, we tested whether cleaners from socially-complex versus simple habitats respond differently to cortisol injections in terms of their cleaning interactions with clients. We found that only cleaners from the socially-complex habitat respond to cortisol injection with strategies functioning as tactical deception: i.e. increased tactile stimulation to small clients and increased cheating of large clients relative to small ones. At the socially-simple site, where reputation management is less important, cortisol-treated fish increased their overall levels of cheating, especially of small clients. Thus, strategic adjustments to cooperative behaviour and tactical deception are likely context-dependent, forming part of general reputation management abilities in cleaner wrasse.

## Introduction

Social interactions are often shaped by communication networks^1^. For instance, bystanders can eavesdrop on ongoing interactions in order to extract information such as fighting ability or aggression levels from future potential interaction partners, and adjust their own responses accordingly^1, 2^. Alternatively, individuals involved in an ongoing social interaction can adjust their behaviour in response to being observed, for example, to appear more aggressive or more cooperative than they would be otherwise. This adjustment of one’s behaviour in response to eavesdropping has been termed audience effects^3^. Depending on the stakes, audience effects may yield an increase in aggression to deter rivals, or an increase in cooperation to attract cooperators^4, 5^.

Audience effects contain, at least in part, false information: individuals present themselves as more aggressive, more cooperative or more harassed than they really are. For instance, low ranking chimpanzees will give exaggerated distress calls if the audience contains an individual that outranks their aggressor and could potentially intervene^6^. In extreme cases, an individual may produce a signal outside of its normal context leading the receiver to respond in a disadvantageous way to the signaler’s benefit, behaviour referred to as tactical deception^7^. In a classic anecdote^8^, a young baboon gave distress calls without having been aggressed near adults holding attractive food. Apparently in response, the mother attacked the perceived aggressor, who fled and dropped the food, which the young baboon then ate. The interpretation of this sequence of events was that the young baboon deliberately produced a signal out of context to fool his mother into helping him by chasing the supposed aggressor^8^. Tactical deception can, thus, evolve as a byproduct of selection for cooperative behaviours^9, 10^. To summarize, eavesdropping in communication networks may lead to diverse audience effects and, hence, sophisticated social strategies.

Currently, we know relatively little about the physiological and/or cognitive mechanisms underlying audience effects, including tactical deception. Hence, the extent to which individuals respond to changes in external or internal states by varying their social behaviour is unclear. Based on anecdotal observations in primates^8, 11^, it was initially proposed that tactical deception requires complex cognitive processes, including a ‘theory of mind’ (the ability to perceive other individuals as agents with own desires and beliefs^12^). Thus, tactical deception was seen as an integral part of the Machiavellian intelligence hypothesis and the social brain hypothesis, which both propose that the complexity of life in social groups selected for large brains^11, 13^. Evidence of tactical deception is, nonetheless, found in a diverse range of taxa. For instance, Amazonian flycatchers (*Lanio versicolor* and *Thamnomanes schistogynus*) and capuchin monkeys (*Cebus apella nigritus*) occasionally give predator alarm calls in the absence of a threat to monopolize food resources from con- and/or heterospecifics^14, 15^. Other forms of tactical deception have been documented in birds^16–18^, primates^8, 19, 20^, antelopes^21^, fish^22, 23^, and cephalopods^24^. Such evidence makes studying the mechanisms underlying various social strategies a key prerequisite for understanding the links between behaviour, cognition and brain evolution. Although humans use theory of mind for tactical deception, other species may learn to tactically deceive through operant conditioning^7^. Alternatively, changes in physiological states may lead to context-dependent tactical strategies^23, 25^. However, few studies have empirically investigated the physiological or cognitive mechanisms underlying when and in what contexts such strategies should be employed, but see refs 23, 26.

Interspecific cleaning mutualisms involving the bluestreak cleaner wrasse, *Labroides dimidiatus*, represent an ideal opportunity to explore the drivers of variation in levels of cooperation and tactical deception across contexts. Cleaners provide a service by eating ectoparasites off the surfaces of heterospecific “client” fishes. Although these interactions are generally mutualistic^27–29^, a variety of potential conflicts of interest exist between *L. dimidiatus* and its clients. Most notably, *L. dimidiatus* prefers eating the mucus of clients over ectoparasites^30, 31^. Such an action constitutes cheating on behalf of the cleaner as mucus serves as a protective layer for fish against abrasions, ultraviolet radiation and pathogen infection^32, 33^. Cheating by cleaners is correlated with the clients’ reaction of a full-body “jolt” of movement in response to surface contact with a cleaner mouth (a bite)^34^. Clients can employ a variety of partner control mechanisms to dissuade cheating depending on their strategic options. For instance, visitor clients with access to several cleaning stations may switch to a different cleaner for their next inspection if cheated, whereas resident clients with access to the local cleaner use punishment through aggressive chasing to incite honest service^35–38^. Furthermore, visitor fish arriving at a cleaning station will eavesdrop to extract information about the local cleaner’s service quality and will only respond with invitation for inspection if the cleaner is seen behaving cooperatively^22, 36^. Despite these disincentives, the temptation to cheat remains high: *L. dimidiatus* gain not only additional calories, but also essential amino acids by eating client mucus^39, 40^. As a result, there is pressure on cleaners to make strategic decisions about how often and which clients they should bite under which circumstances in order to maximize their gain.

Service quality can vary greatly between and within individual cleaners. For example, normally cooperative individuals can temporarily become ‘biting cleaners’ that selectively cheat large non-predatory clients, in particular visitors^22^. Such individuals can experience reputational problems as cheating can be observed by bystanders, which will not typically allow a cleaner to approach after witnessing a cheating event. Biting cleaners can improve their reputation by providing small, resident clients with tactile stimulation (TS) from their pelvic and pectoral fins^22^, an action that reduces stress in clients^28^. Functionally, provisioning of TS to small clients in this circumstance works as tactical deception as is used outside of its normal context: TS is typically used for reconciliation and manipulation of current client decisions^41^. When performed on small residents, TS incites passing larger clients to invite inspection by cleaners, often to their own detriment and to the biting cleaners’ advantage. As tactical deception is generally employed by females, mostly in the spawning season, it is likely used to maximize energy gain when energetic demands are high^42^. Indeed, a physiological basis for cleaner’s switch from cooperation to cheating has recently been demonstrated experimentally. Soares *et al*.^23^ injected hydrocortisone (cortisol) into female cleaners to simulate the fish’s stress response, which is the outcome of the activation of the hypothalamic-pituitary-interrenal (HPI) axis, and induce a state of high energy demand^43^. Hormone-treated cleaners provided more TS to small clients and more bites to large clients, consistent with what is expected if cleaners are employing tactical deception^23^. Yet, whether cleaners choose to employ this strategy based on learned decision rules, or simply respond behaviourally to a physiological state of energetic need, is unclear.

To better understand the mechanisms underlying strategic adjustments to levels of cooperation in cleaner wrasse, we took advantage of recent findings suggesting that the interspecific social complexity of a habitat predicts the extent to which ‘normal’ cleaners care about their reputation (i.e. take the audience into account^44, 45^). Earlier research demonstrating that cleaners respond to the presence of image scoring bystanders with increased levels of cooperation had invariably used cleaners caught from reef habitats with high levels of social complexity^36, 37^. In contrast, Wismer *et al*.^44^ found that cleaners from a low social complexity reef, characterized by low client density and richness as well as low competition between cleaners over access to clients, typically did not show such audience effects. Although explanations for these observed differences in strategic behaviour are lacking, some evidence suggests that both the opportunity and need to learn are lower in cleaners from habitats with low social complexity (Wismer *et al*. unpublished data). In any case, these observed differences allowed us to test whether cortisol injections cause patterns of strategic behavioural adjustment consistent with tactical deception independently or as a function of everyday reputation management strategies. If reputation management is a prerequisite for such strategies, we hypothesize that strategic adjustments to baseline levels of cooperation should be employed by cortisol-injected cleaners in high complexity habitats only. If cleaners are engaging in tactical deception, we predict that cortisol-injected cleaners from high complexity habitats should provide more TS to small clients, and more bites to large clients. Conversely, cortisol-injected cleaners from low-complexity habitats should not adjust their behaviour based on client size, but should cheat more overall (i.e. give more bites to all clients) in response to their increased state of stress.

## Results

### Reef surveys

**Figure 1:**
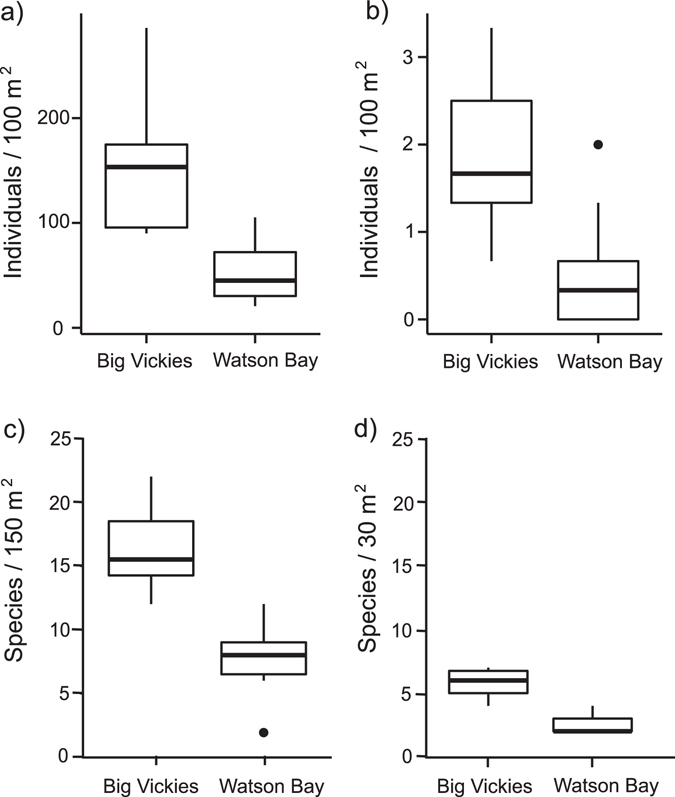
Individual and species counts at the two study sites. (**a**) Boxplots (median and interquartile range) of client fish abundance (number of individuals) and (**b**) cleaner wrasse (*L. dimidiatus*) abundance estimated over 100 m^2^ from 10 transects each at Big Vickies (high social complexity) and Watson Bay (low social complexity) sites around Lizard Island. (**c**) Boxplots (median and interquartile range) of large (species >10 cm TL, estimated over 150 m^2^) and (**d**) small (species <10 cm TL, estimated over 30 m^2^) client species richness (number of species excluding *L. dimidiatus*) from same transects as above.

Our two study sites differed in four measures of social complexity (Fig. 1). The high-complexity site (Big Vickie’s) had higher client species abundance (Mean per 100 m^2^ ± SEM: 158.4 ± 22.8 vs. 52.6 ± 9.0; N = 20, t = 5.11, *p* < 0.0001, CI = 0.29–0.71), cleaner wrasse abundance (Mean ± SE: 1.8 ± 0.3 vs. 0.5 ± 0.2; N = 20, t = 3.55, *p* = 0.0025, CI = 0.51–2.01), large client richness (Mean per 150 m^2^ ± SEM: 16.2 ± 0.97 vs. 7.9 ± 0.9; N = 20, GLM: χ^2^ = 29.2, *p* < 0.0001, *pseudo R* ^*2*^ = 0.65) and small client richness (Mean per 30 m^2^ ± SEM: 5.8 ± 0.3 vs. 2.6 ± 0.3; N = 20, GLM: χ^2^ = 12.5, *p* = 0.0004, *pseudo R* ^*2*^ = 0.76) than the low complexity site (Watson Bay).

### Baseline cortisol levels

Cleaners sampled for analysis of baseline cortisol levels ranged in size from 6.6–7.7 cm (Mean ± SE: 7.0 ± 0.1 cm SL) on low complexity reefs and 5.7–7.9 cm (Mean ± SE: 7.2 ± 0.2 cm SL) on high complexity reefs. Whole body cortisol levels in cleaners did not differ significantly between these reef types (N = 21, t = 0.243, *p* = 0.81, Supplementary Fig. S2).

### Laboratory image scoring experiments

#### Tactile stimulation

**Figure 2:**
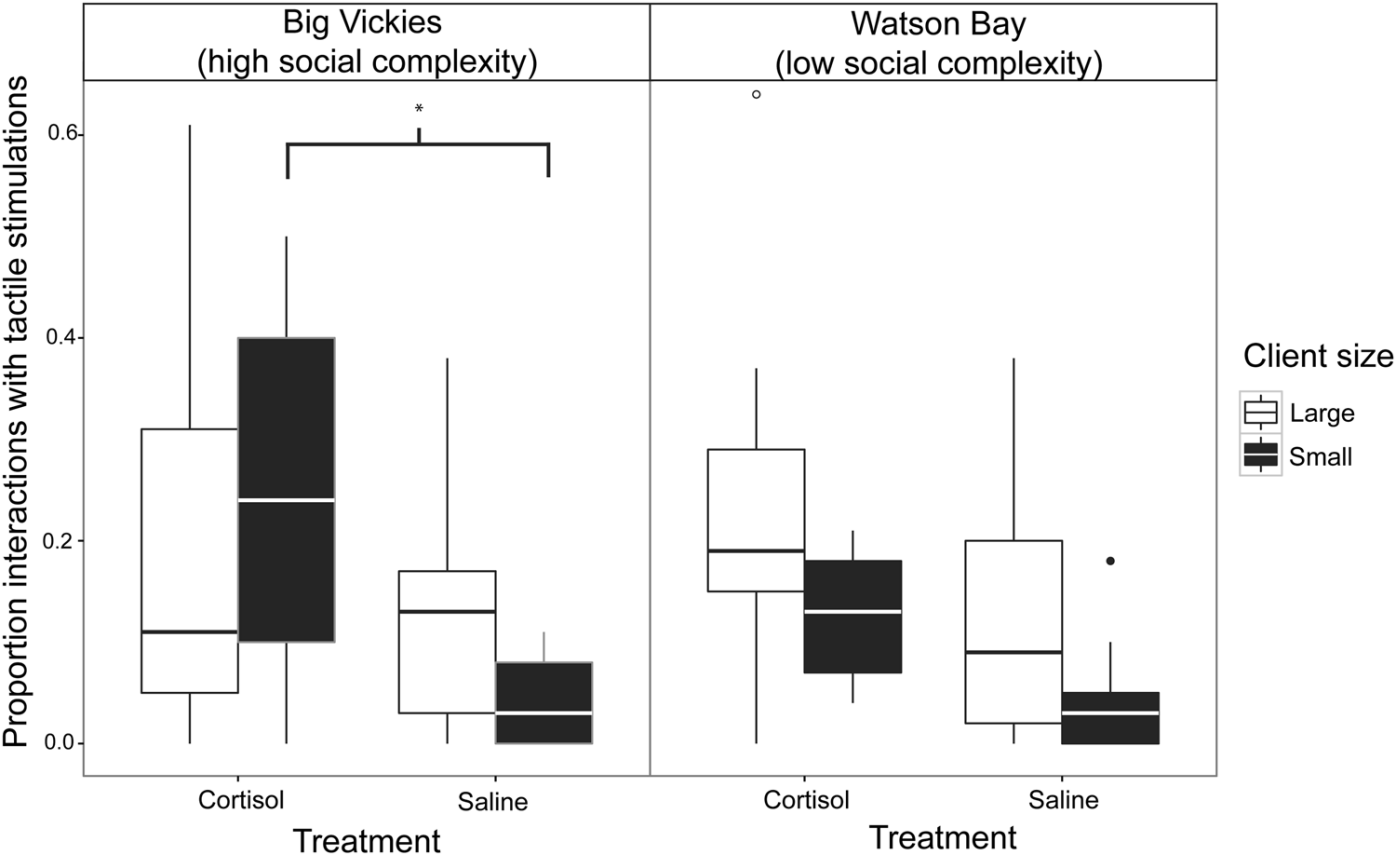
Proportion of interactions with client fish involving tactile stimulations. Boxplots (median and interquartile range) showing proportion of interactions with clients that included tactile stimulation for cleaner wrasse injected with cortisol or saline, at high (Big Vickies) or low (Watson Bay) social complexity sites, and interacting with large or small clients. See Figure S3 for model predictions and effects plots depicting the significant difference between the proportion of interactions with tactile stimulations given by cortisol and saline treated cleaners to small clients at high-complexity sites.

Our model explained almost 25% of the variation in TS observed (*R* ^2^_GLMM(m)_ = 0.247, *R* ^2^_GLMM(c)_ = 0.367). The three-way interaction among site, treatment and client body size was marginally significant (N = 2 177 client interactions, GLMM: χ^2^ = 3.86, *p* = 0.049; Fig. 2, Supplementary Table S1): Cortisol-treated cleaners gave more TS to small clients than saline-treated cleaners but only in high social complexity habitats (Supplementary Figure S3). Results from parametric bootstrapping showed that the three-way interaction term explained only a marginally significant proportion of the residual deviance when compared to the full model lacking this term (PBtest: LRT = 3.766, *p* = 0.052, 1000 simulations). We therefore report significant lower-order terms as well. There was a significant two-way interaction between client size and site (χ^2^ = 8.61, *p* = 0.003, Supplementary Table S1): large clients receive more TS than small clients in sites with low social complexity. The main effect of treatment on the occurrence of TS was significantly different between cortisol and saline-injected fish (χ^2^ = 14.29, *p* = 0.0002) with cortisol-treated cleaners giving TS to clients in a higher proportion of interactions than saline-treated fish. There was also a positive relationship between interaction duration and the occurrence of TS (χ^2^ = 81.217, *p* < 0.0001).

#### Jolts

**Figure 3:**
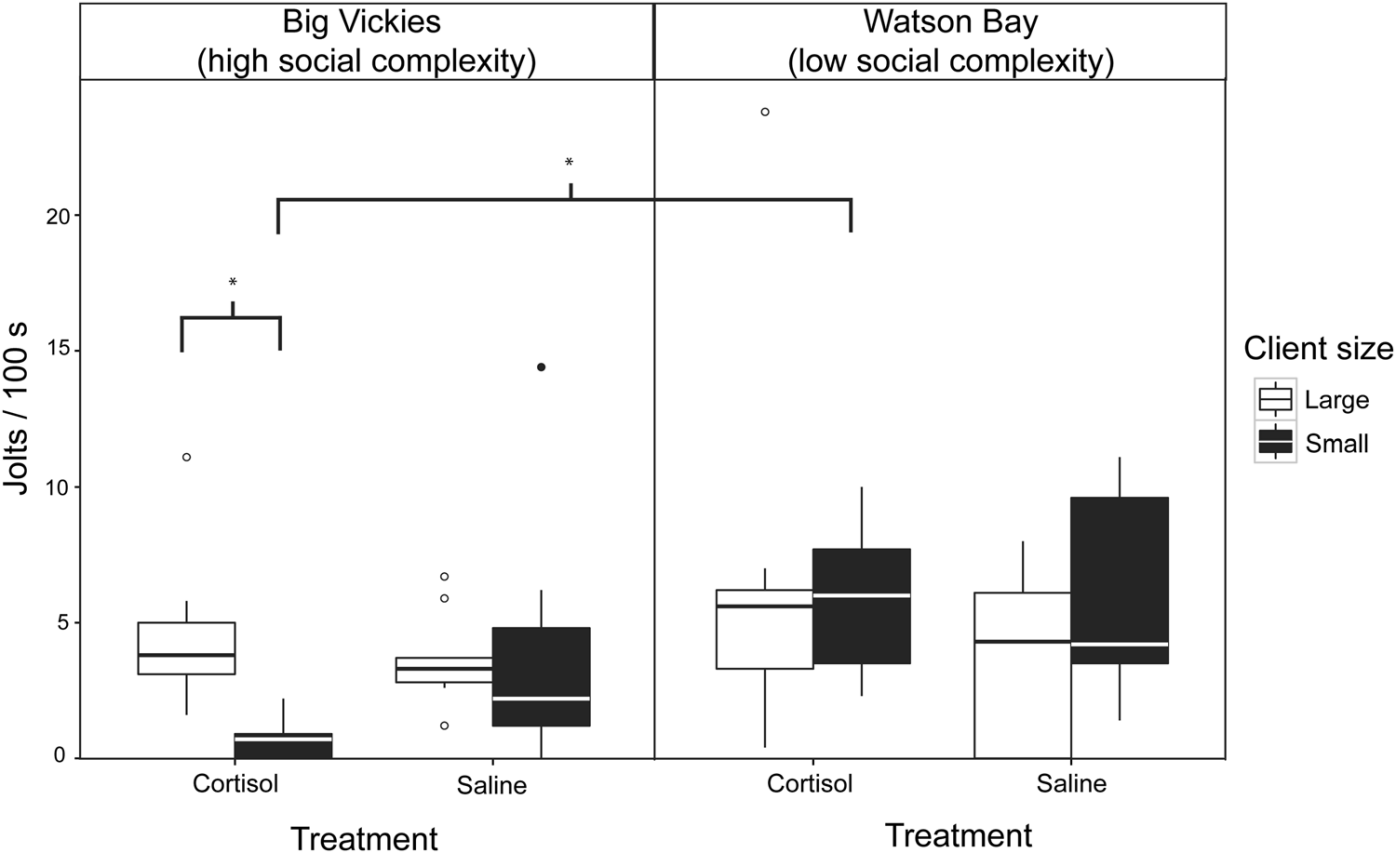
Number of jolts (cleaner bites) per 100 seconds of interaction time with clients. Boxplots (median and interquartile range) showing mean number of jolts (scaled to interaction durations of 100 s) for cleaner wrasse injected with cortisol or saline, at high (Big Vickies) or low (Watson Bay) social complexity sites, and interacting with large or small clients. See Figure S4 for model predictions and effects plots depicting the significant difference between jolts received by small and large clients at high-complexity sites by cortisol-injected cleaners and S5 for the significant difference in jolts received by small clients from cortisol treated cleaner wrasse at sites of low and high social complexity.

Fixed factors in our model explained approximately 11% of the variation in number of client jolts observed (*pseudo R* ^2^ = 0.109). There was a significant three-way interaction among client size, treatment and site (N = 2 180 client interactions; GLMM: χ^2^ = 7.238, *p* = 0.007; Fig. 3, Supplementary Table S2). In the high social complexity site, cortisol-treated cleaners caused large clients to jolt more often than small clients (Supplementary Fig. S4). In contrast, cortisol-treated cleaners caused small clients to jolt more than larger clients in the low complexity site (Supplementary Fig. S5). Although there was a tendency at the high complexity site for larger clients to receive more jolts from cortisol-injected than saline-injected cleaners, this difference was not significant (Fig. 3). Results from parametric bootstrapping showed that the three-way interaction term explained a significant proportion of the residual deviance when compared to the full model lacking this term (PBtest: LRT = 7.578, *p* = 0.0059, 1000 simulations). Jolt frequency increased significantly with interaction duration (GLMM: χ^2^ = 28.73, *p* < 0.0001, Supplementary Table S2).

## Discussion

The rationale of our experiments were based on three previous studies: the documentation of tactical deception of clients by cleaner wrasse^22^, the demonstration that injection of cortisol can trigger increased occurrence of tactical deception^23^, and the observation that cleaners from high versus low social complexity habitats differ in their reputation management strategies^44^. By subjecting cleaners from both habitats to cortisol injections, we could ask whether cortisol invariably triggers behavioural changes that are functionally tactical deception, or whether a cleaner’s response to increased cortisol depends on other variables. Our results show that only cleaners from the habitat with high social complexity respond to cortisol injection with strategies functioning as tactical deception, i.e. increased tactile stimulation to small clients and increase cheating of large clients relative to small ones. This was achieved through a reduction in the number of bites given to small clients rather than an increase in the overall number of bites given to large clients as we had predicted. The differences between cleaners from the high and low social complexity site cannot be explained by different basal cortisol levels leading to different end concentrations of cortisol due our injections (Supplementary Fig. S2). Thus, there is no direct causal link between cortisol concentrations and the occurrence of selective changes in service quality in cleaner wrasse. Instead, cortisol injections signal a change of energetic need and individuals must decide how to respond to meet these requirements. We hypothesized that reputation management strategies may affect the behavioural response of cleaners to cortisol injection. Indeed, our results are broadly consistent with this hypothesis: only cleaners from the high social complexity site, where cleaners typically respond to image scoring clients with audience effects (i.e. increased service quality)^44, 45^, displayed patterns consistent with tactical deception when injected with cortisol. In contrast, cleaners from the low social complexity habitat, where evidence for audience effects under normal conditions is lacking^45^, did not strategically adjust TS or cheating rates to different client types, but cheated more frequently, especially on smaller clients (Supplementary Fig. S4). We note that cortisol injections at the high-complexity site seemed to reduce jolts in small clients more than it increased jolts in large clients (Fig. 3). Thus, it is not clear from our data that the changes in behaviour observed in cortisol-injected cleaners represent a cost to large clients relative to the baseline levels of cheating occurring in the controls. Furthermore, we could not explicitly test the frequency with which TS given to small residents is immediately followed by a jolt given to a large client due to the short time window for observations following manipulations (45 min per cleaner). The increased proportion of TS given to small clients at the high complexity site may, indeed, entice large clients to approach the cortisol-injected cleaners. However, this strategy may simply be a way for cleaners to access image-scoring clients in situations when energy needs are high rather than to deceive per se. Nonetheless, the significant three-way interaction confirms the differences in strategic adjustments between these two sites.

It is important to realize that the observed link between reputation management and strategic behavioural adjustments are only correlational. Furthermore, we only compare two sites. Although we interpret the behavioural differences observed between these sites to be due to differences in their social complexity, other explanations exist. For instance, cleaners from the two sites may differ in various other ways, including physiologically (i.e. different amount of gluccocorticod receptors in their brains and/or responsiveness to increases in cortisol). Differences could also be directly caused by intrinsic habitat differences (i.e. water currents, habitat quality). For example, Wismer *et al*.^44^ reported a lower overall frequency of interactions, especially with large visitor clients, in habitats with low social complexity. Thus, situations in which strategic behavioural adjustments yield benefits (i.e. a large potential client arriving at the cleaning station and inviting inspection *because* it sees a small client receiving high quality service) may simply be too infrequent at these sites for cleaners to benefit much by adjusting to them. Although we did not observe differences in the number of interactions with small and large clients between sites or injection treatments (Supplementary Table S3), evidence from field observations suggests that relevant situations, such as cleaners having to choose between residents and visitors seeking cleaning simultaneously, are generally much rarer when social complexity is low (Wismer *et al*. unpublished data). However, laboratory experiments suggest that cleaners from low social complexity habitats are not capable of adjusting to image scoring even when it would be beneficial to do so^44, 45^. Also, the circumstances leading to increased service quality or to tactical deception in cleaners from high complexity habitats are similar: both involve image scoring bystander clients and cleaners showing behavioural adjustments in response. We therefore propose that cleaners from high complexity habitats learn to respond to image scoring in a flexible way, i.e. as a function of their internal physiological state. A ‘normal’ physiological state leads to overall increased levels of cooperation while high energetic demands lead to strategies which may function as tactical deception. Mathematical modelling shows that such state dependent decision making can be adaptive^25^.

We currently lack an understanding of the mechanisms underlying audience effects, including behaviours qualifying functionally as tactical deception. Our study provides insights into the mechanisms behind context-dependent strategic behavioural adjustment in cleaner wrasse: we can exclude the simplest potential explanation, i.e. a physiological agent directly causing the behavioural patterns. Instead, while previous studies have suggested that energetic needs alone seem to cause more cheating, individual cleaners appear to decide how to implement cooperative behaviours versus cheating with a variety of clients within a communication network. In this context, the general ability to manage one’s own reputation emerges as a prime candidate for the expression of strategic behavioural adjustment that may function as tactical deception. Its expression is likely due to individual flexibility in decision making. While the current study cannot address whether cleaners understand how their reputation works, nor the direct costs of deceptive strategies to receivers, our results do show that cleaner wrasse are able to fine-tune their behaviour in sophisticated ways to both internal and external circumstances.

## Methods

All methods of fish capture, handling, injection, euthanasia and observations were approved and performed in accordance with the relevant guidelines and regulations outlined by the Government of Queensland’s Department of Agriculture and Fisheries Animal Ethics Committee (approval numbers CA 2014/07/782 and CA 2015/06/869).

### Study sites and reef surveys

This study was conducted at the Lizard Island Research Station (LIRS, 14° 40′S, 145° 28′E) Northern Great Barrier Reef, Australia between July and September 2014. Two sites near the Lizard Island Research Station, Australia were selected for field experiments. A high social complexity site (Big Vickie’s) was chosen on the southern edge of Lizard Island. This area of contiguous reef is approximately 90,000 m^2^ ranging in depth from 1–10 meters. A low social complexity site (Watson Bay) was selected in a protected bay on the western part of the island. This site comprises a series of isolated reef patches measuring 2 to 10 meters in length at about 6–7 meters in depth interspersed by 1 to 7 meters of open sand areas. We used 30 m belt transect surveys to estimate social complexity at each site^44, 46^. One scuba diver swam with a transect tape across the reef at a constant speed, and recorded the number and species identity of all individuals greater than 10 cm in length within 2.5 m on either side of the tape (large clients; 150 m^2^). A second diver followed behind the first to verify transect length. Once 30 m was reached, the two divers then swam back along the tape recording the abundance and species identity of all individuals smaller than 10 cm within 50 cm on either side of the tape (small clients; 30 m^2^). Having the second diver lying out the transect tape closely behind the first has been shown to reduce diver effects^47^. Ten transects were recorded per reef site covering all representative microhabitats at each site (1 500 m^2^ of habitat surveyed per site). All transects were conducted by the same diver team (OR and SW) on relatively calm weather days between 9:00 and 15:00 in July and August when visibility was good to standardize the fish counts as much as possible. Total client and cleaner wrasse (*L. dimidiatus*) abundance was estimated for each transect and standardized to an area of 100 m^2^.

### Baseline cortisol levels in cleaner wrasse

We compared whole body cortisol levels from adult female cleaner wrasse collected from sites differing in social complexity around Lizard Island. Seven adult female cleaners from patches at Watson Bay were collected in July-August 2015. As fish collection for the purpose of tissue extraction is not permitted at the Big Vickie’s site, we collected 14 individuals from nearby socially-complex reefs that have been used in earlier studies documenting audience effects. Fish were collected using a small monofilament barrier net (2 m H × 1 m L, 10 mm stretch mesh) and a hand net, and transported directly to a waiting researcher (ZT) on the boat for immediate processing. Fish were sacrificed by cervical transection and the bodies were kept on ice during transport back to the LIRS facilities. Samples were then frozen at −20 °C, and shipped to the University of Neuchâtel in Switzerland for further processing.

In Neuchâtel, fish bodies were weighed and placed in a volume of 100% methanol equivalent to nine times the tissue weight. The mixture was then homogenized using a VWR® 200 Homogenizer, and 1 mL of the homogenized mixture was centrifuged at 3007 *g* and 4 °C for 5 min. The supernatant was dried in a vacuum pressure Speedvac®, then reconstituted with 990 uL of 5% methanol solution and spiked with 10 uL of an 80 ng/mL solution of isotopically labelled internal standard (compound ref. Cortisol-9,11,12,12-d4, TRC). Extracts were passed through solid-phase extraction (SPE) cartridges and further partitioned by liquid-liquid extraction (See Supplementary Methods for details). The final extracts were analysed by UHPLC-MS/MS (See Supplementary Methods for details). Cortisol levels were expressed as ng per 1 gram of tissue weight.

### Cortisol injections and behavioural observations

Methods of cortisol preparation, injection and observation followed Soares *et al*.^23^. To prepare the cortisol solution for injection, 0.05 g of hydrocortisone ≥98% (HPLC, Sigma Laboratories) was dissolved in 1 ml of 96% ethanol solution and left overnight to evaporate. Cortisol was first diluted in a 10 ml saline solution (Sodium chloride, injection BP 0.9%, 45 mg in 5 ml) and then 102 µl from this cortisol solution was further diluted in 5000 µl of saline to obtain a stock solution of 1 µg/ml. Control saline solutions were prepared in the same way excluding the addition of hydrocortisone. Both solutions were stored at 4 °C for the duration of the experiments.

All manipulations and behavioural observations were conducted underwater between 8:30 and 17:00 at depths of 1–9 m by two to three SCUBA divers. As cleaner wrasse typically spawn between October and December, all observations were conducted during the non-spawning season. Eighteen female cleaner wrasse were randomly caught using a monofilament barrier net at each site for observation (nine fish per treatment per site). Fish total length (TL) was measured to the nearest cm using a flexible ruler to determine injection volume based on known length-mass relationship for this species (see Supplementary Fig. S1). Fish were then injected intramuscularly with either saline solution or cortisol solution corresponding to 1 µg per g of body mass. Injection volumes ranged from 20 µl to 40 µl. Treatment order was counterbalanced within and between sites. Fish were released following injection and filmed by a diver for 45 min with Canon G15 cameras from a distance of approximately 2 m. No fish suffered from detectable injury or death after the injections or behavioural observations. All methods of fish capture, handling, injection and observations were approved by the Government of Queensland’s animal ethics committee.

### Video analyses

Video recordings were analyzed by the same researcher (OR), who was blind to the site and treatment of each video at the time of analysis (videos were renamed by a third party prior to analysis). For each video, OR recorded a) client fish family; b) client fish size (TL estimated visually to the nearest cm); c) the duration (seconds) of each interaction d) whether tactile stimulation (when a cleaner touches the body of the client with its ventral region and pelvic fins in the absence of feeding) was provided; and e) the number of jolts (whole-body shudders in response to cleaner mouth contact) performed by the client during each interaction. Jolts are good correlates of biting (cheating) in clients as they typically occur when cleaners ingest client mucus and/or scales as opposed to ectoparasites^34^. We distinguished two client size categories as in Soares *et al*.^23^. Small clients were <14 cm TL and consisted mostly of resident individuals from the following families: Chaetodontidae, Pomacentridae, and Labridae. Large clients measured >14 cm TL and were predominantly Acanthuridae, Caesionidae, Labridae and Nemipteridae, which tend to be roving visitors at cleaner stations. Client size has previously been used as a proxy of cleaner wrasse food value with larger clients considered higher value^48, 49^.

### Statistical analyses

We performed statistical analyses using R 3.1.2. From our reef transect surveys, we compared the abundance and diversity of client fish and the abundance of cleaner wrasse between our two study sites using Student’s t-tests. Baseline levels of cortisol between reef types (high vs. low social complexity) were also analyzed using a Student’s t-test. We tested for homogeneity of variances and normality using Fisher’s F-tests and Shapiro-Wilkes tests. Client abundance and whole body cortisol levels were log10-transformed to meet model assumptions.

Differences in large and small client species richness between the two study sites were tested using generalized linear models (GLM) with a poisson error distribution using the glm function in the R package “lme4”^50^. We tested for overdispersion using the dispersiontest function in the R package “AER”^51^. Normality of residuals were verified using qqplots. We calculated a pseudo *R* ^2^ by comparing the residual deviance by the null deviance of the model (*pseudo R* ^2^ = 1 − (residual deviance / null deviance)).

We recorded 2180 interactions from 36 cleaners. From these videos, we scored all occurrences of cheating behaviour and tactile stimulations. We used a generalized linear mixed model (GLMM) with a binomial error distribution (glmer function in the package lme4^50^) to test whether the occurrence of tactile stimulations was affected by hormone treatment, habitat social complexity (site) and/or client fish size (fixed factors) while controlling for cleaner identity and client family (random factors). Interaction duration was centered and Z-standardized using the “scale” function^52^, and included as a covariate. We excluded interaction durations longer than 100 seconds as these occurred infrequently (3 occurrences in 2180 interactions). Excluding these values did not qualitatively change the model results, but improved diagnostics. We included two-way interactions between client size, treatment and site and a three-way interaction among these factors. There was no significant difference ( *p* > 0.10) between models with and without 2-way interactions between main effects and duration, and the model without interactions between main effects and duration had a lower AIC score than models which included these additional two-way interactions. Normality of residuals for random factors were verified using qqplots. We calculated the marginal *R* ^2^ (variance explained by the fixed factors; *R* ^2^_GLMM(m)_) and conditional *R* ^2^ (variance explained by the fixed and random factors; *R* ^2^_GLMM(c)_) following Nakagawa and Schielzeth^53^ using the “MuMIn” package^54^. We used the “effects” function from the R package “effects”^55^ to visualize interactions. We also performed a parametric bootstrapping model comparison (100 bootstrapped samples) using the function “PBmodcomp” from the package “pbkrtest”^56^ to assess the significance of the three-way interaction (Site X Treatment X Size) with a likelihood ratio test.

We used a GLMM with a negative binomial error distribution (glmer.nb function in the R package “lme4”) to test whether cheating rate (number of client jolts) was affected by hormone treatment, habitat social complexity (site) and/or client fish size (fixed factors) while controlling for cleaner identity and client family (random factors). Scaled interaction duration was included as a covariate (all 2180 data points included). As above, we only included two-way interactions between client size, treatment and site and a three-way interaction among these factors because there was no significant difference (*p* > 0.66) between models with and without 2-way interactions between main effects and duration, and the model without interactions between main effects and duration had a lower AIC score. Normality of residuals for random factors were verified using qqplots. We calculated a pseudo *R* ^2^ by comparing the residual deviance by the null deviance of the model (*pseudo R* ^2^ = 1 − (residual deviance/null deviance)). We used the “effects” function from the R package “effects”^55^ to visualize interactions. As above, we performed a parametric bootstrapping model comparison to assess the significance of the three-way interaction (Site X Treatment X Size) with a likelihood ratio test.

### Data Availability

The data for this study will be publicly archived in the repository figshare upon acceptance of the manuscript (doi:10.6084/m9.figshare.3817392) following best practices^57^. Reviewers can access the data here: https://figshare.com/s/28421593a0d49d4bb2c5.

## Electronic supplementary material

Supplemental material
