# Supplementary material for: Reputation management promotes strategic adjustment of service quality in cleaner wrasse
Source: Sci Rep. 2017 Aug 21;7:8425. doi: 10.1038/s41598-017-07128-5 (PMC5566447; doi:10.1038/s41598-017-07128-5)
Supplement: Supplementary file 1 — Supplemental material [file 41598_2017_7128_MOESM1_ESM.pdf]

Electronic Supplementary Material

Reputation management promotes strategic adjustment of service quality in cleaner wrasse

Sandra A. Binning[a], Olivia Rey[a], Sharon Wismer[a], Zegni Triki[a], Gaétan Glauser[b], Marta C. Soares[c], Redouan Bshary[a,*]

[a] Université de Neuchâtel, Institut de Biologie, Eco-Ethologie, Rue Emilie-Argand 11, 2000 Neuchâtel, Switzerland

[b] Université de Neuchâtel, Institut de Chimie, Neuchâtel Platform of Analytical Chemistry, Avenue de Bellevaux 51, 2000 Neuchâtel, Switzerland

[c] CIBIO, Centro de Investigação em Biodiversidade e Recursos Genéticos, Universidade do Porto, Vairão, Portugal

[*] Corresponding author email address: sandra.binning@unine.ch

Supplemental Figures

Figure S1. Relationship between fish total length (cm) and body mass (g) for *L. dimidiatus* collected in 2010 from patch and continuous reefs around Lizard Island (Wismer et al. 2014). Fish were measured in the aquarium facilities at the Lizard Island Research Station. The length-mass regression presented on this figure was used to determine the cortisol concentrations used for injection in the field.

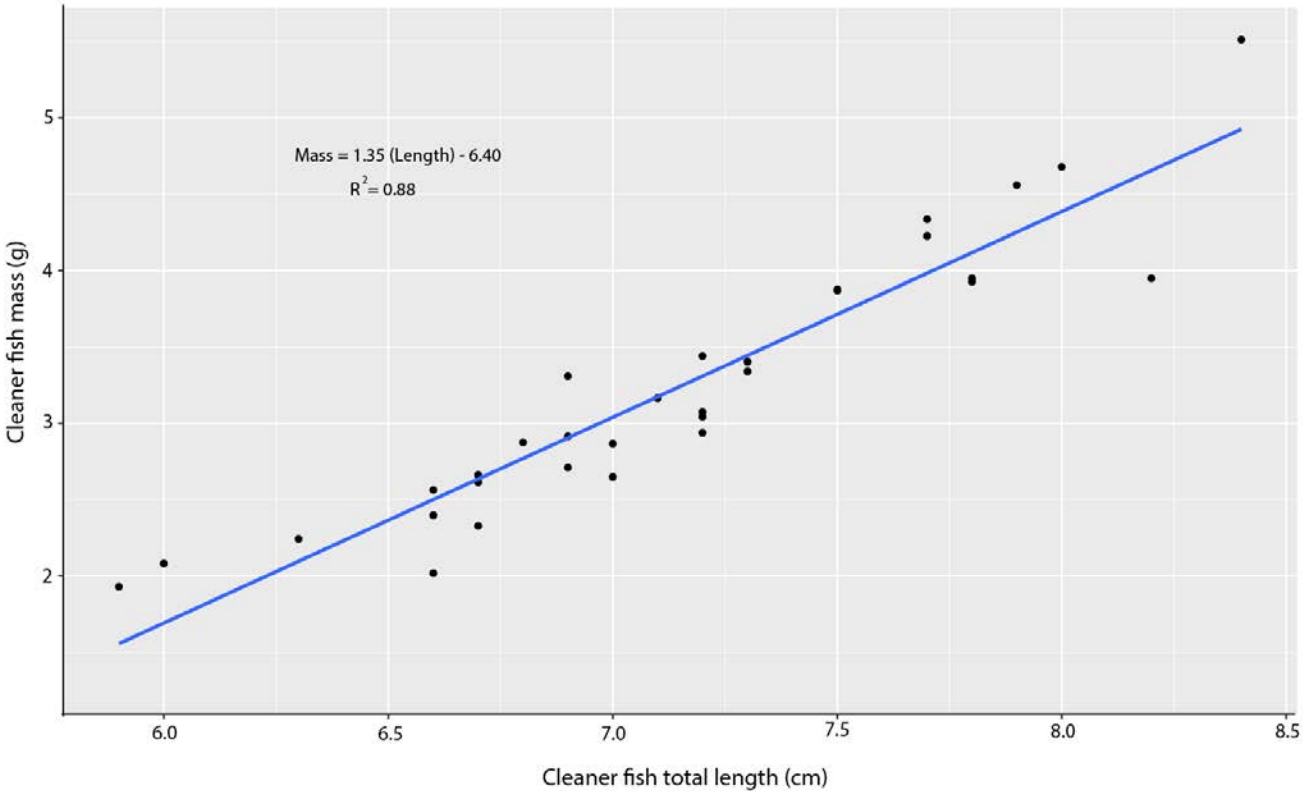

Figure S2. Boxplots (median and interquartile range) of cortisol concentrations (ng/ 100 g fish tissue) extracted from body tissues of wild-caught fish collected from sites with high and low social complexity.

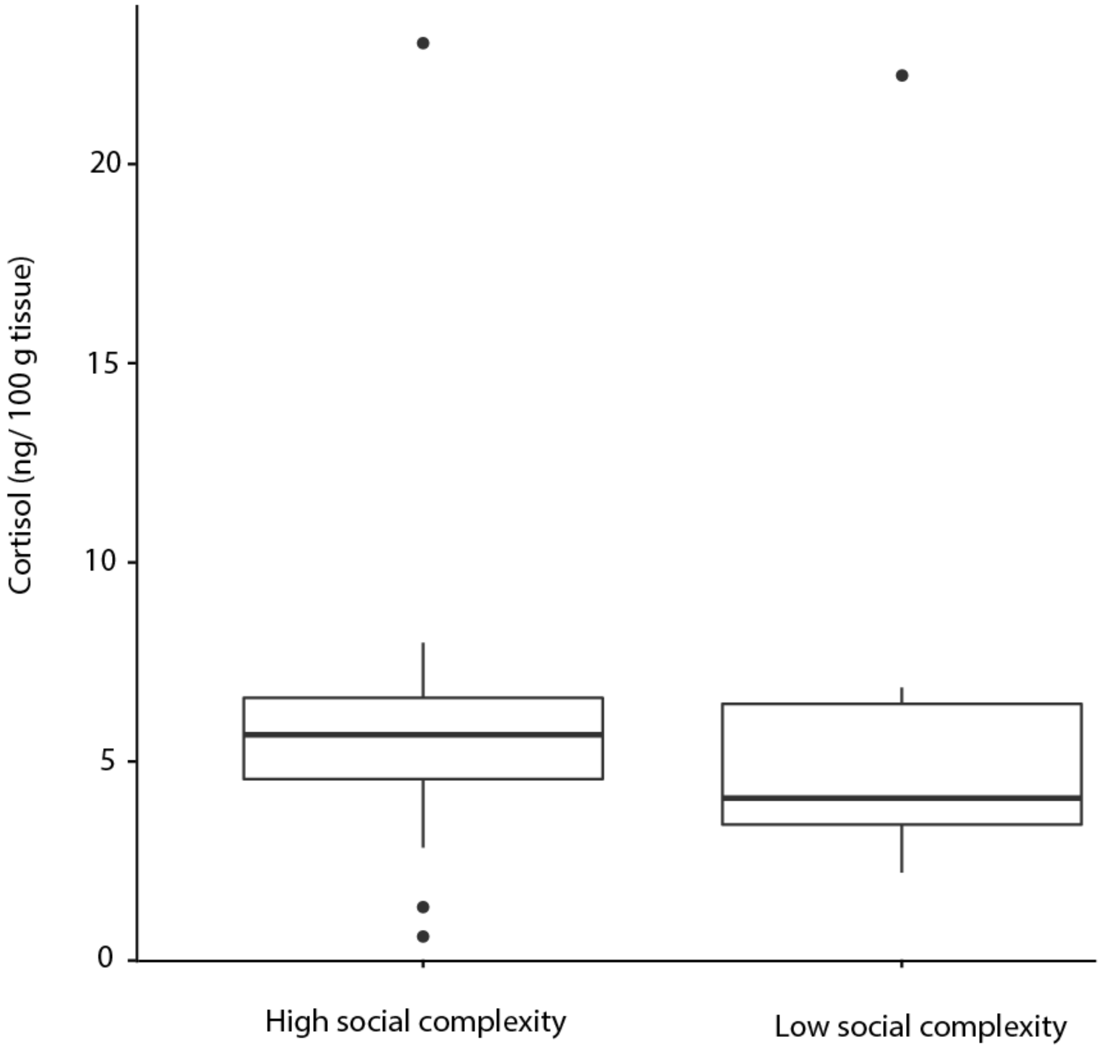

Figure S3. Effects plot of model predictions (parameter estimate and 95% CI) of the three-way interaction among client size, injection treatment and site (interaction duration was specified as a predictor in the model). The top right panel (b) depicts the significant difference between the proportion of interactions with tactile stimulations given by cortisol and saline treated cleaners to small clients at high-complexity sites. All other contrasts had overlapping CI's.

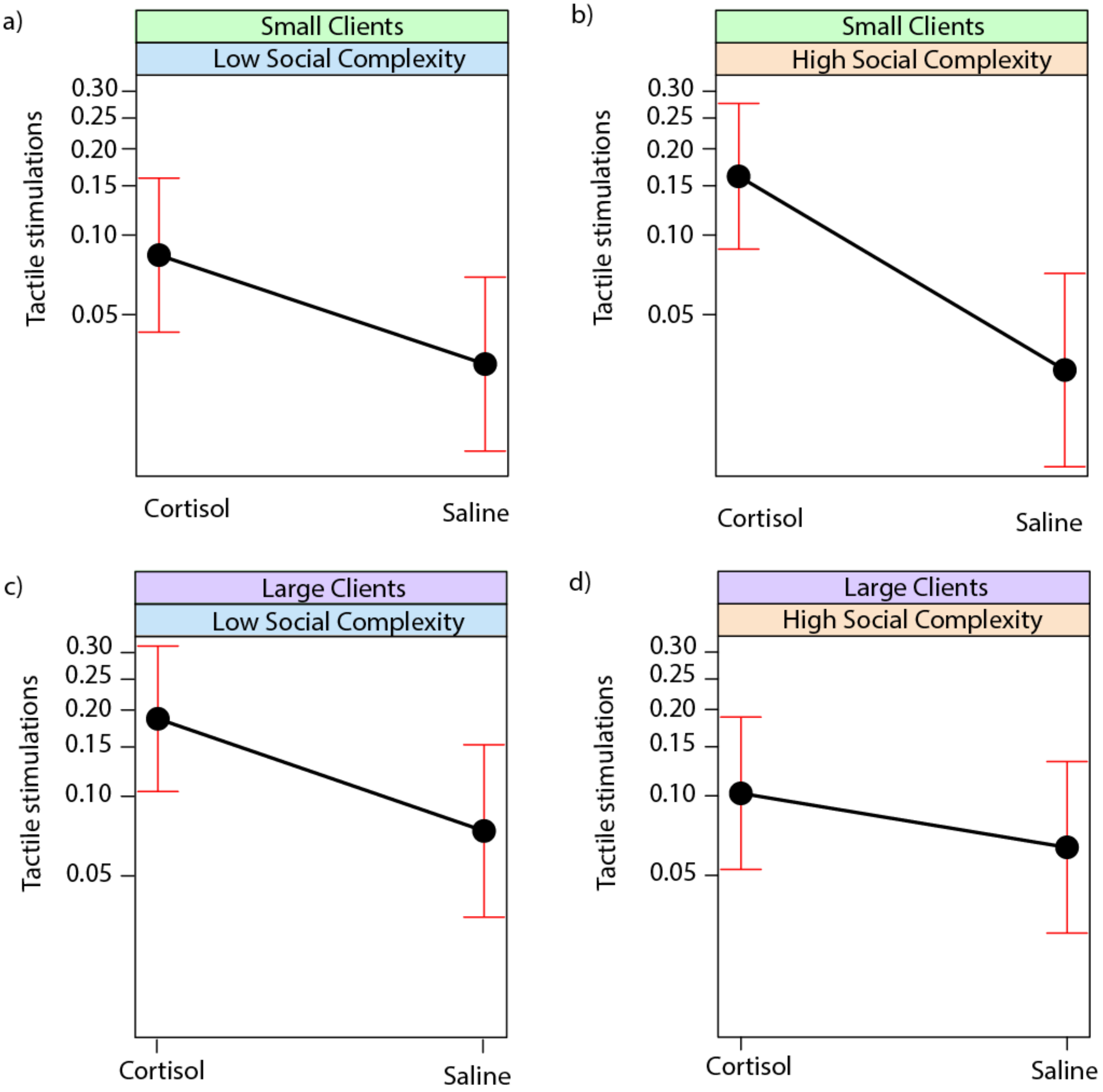

Figure S4. Effects plot of model predictions (mean and 95% CI) of the three-way interaction among client size, injection treatment and site (interaction duration was specified as a predictor in the model). The top left panel (a) depicts the significant difference between jolts received by small and large clients at high-complexity sites by cortisol-injected cleaners.

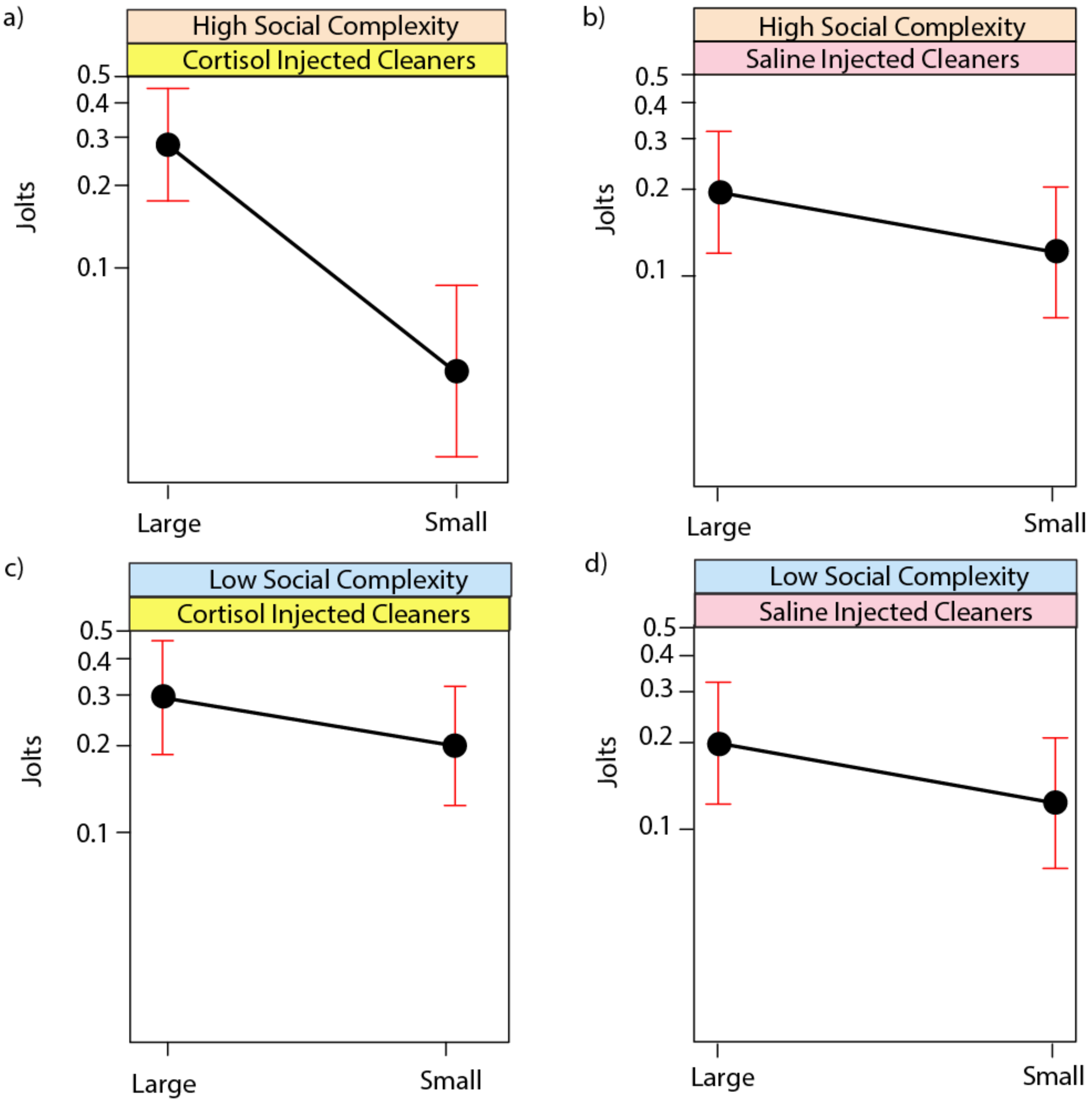

Figure S5. Effects plot of the three way interaction depicting the significant difference in jolts received by small clients from cortisol treated cleaner wrasse at sites of low and high social complexity (top left panel a).

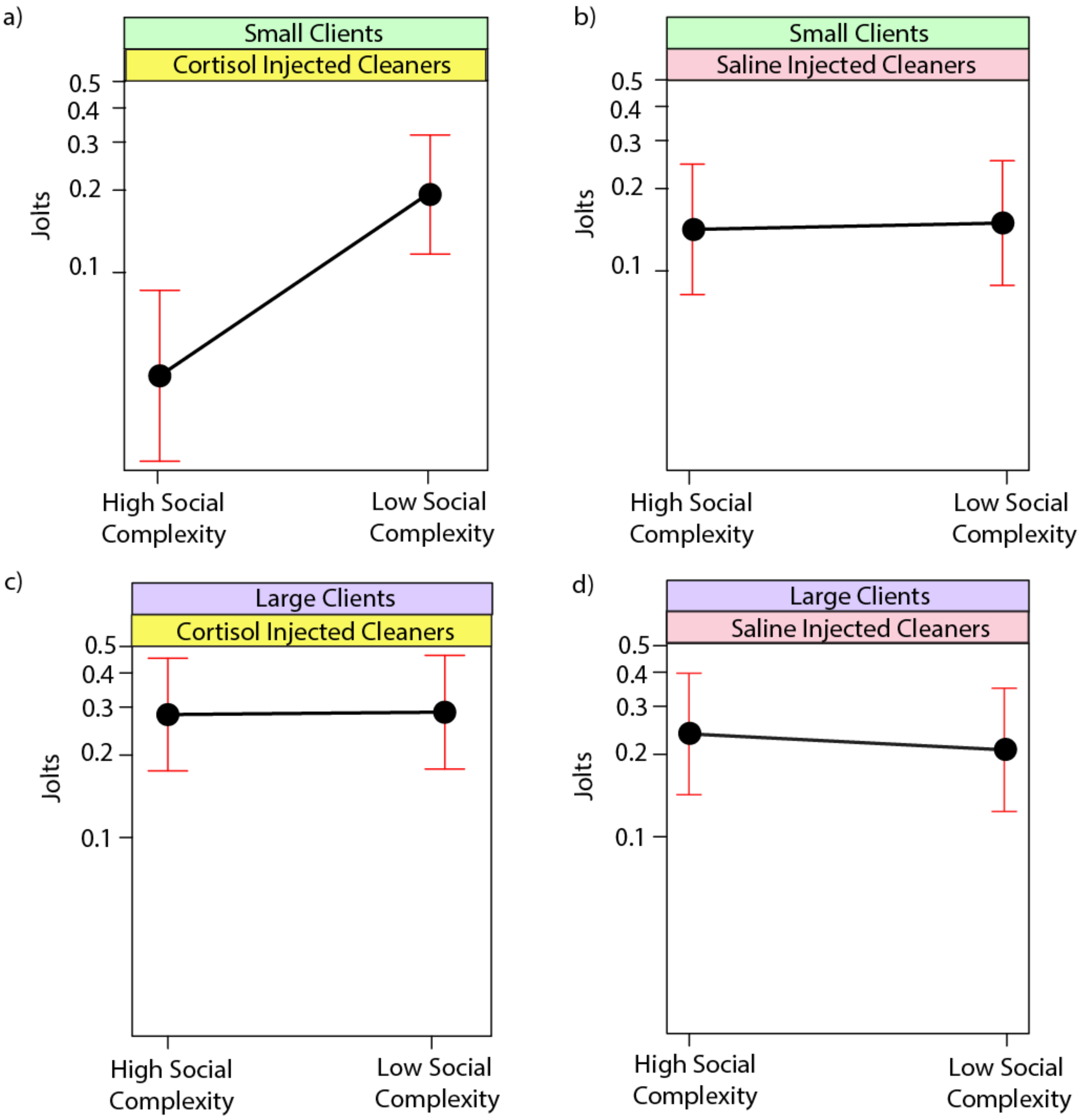

Supplemental Tables:

Table S1: Analysis of covariance table: GLMM model for cleaning interactions with tactile stimulations. Client size (size; large or small), injection treatment (Treatment; saline or cortisol), site (Big Vickies or Watson Bay) and scaled interaction duration (Duration: time spent interacting with client in seconds) were included as main effects. All interactions included in the model are presented. Asterisks depict significant effects ($\alpha = 0.05$).

**Source of variation**	χ^2	**P**
**Main Effects**		
Size	1.867	0.172
Treatment	14.427	0.0001 *
Site	0.123	0.726
Duration	81.217	< 0.00001 *
**Two-way interactions**		
Size X Treatment	2.714	0.099
Size X Site	9.352	0.002 *
Treatment X Site	0.065	0.799
**Three-way interaction**		
Size X Treatment X Site	3.865	0.049 *

Table S2: Analysis of covariance table: GLMM model for number of jolts observed per cleaner interaction. Client size (size; large or small), injection treatment (Treatment; saline or cortisol), site (Big Vickies or Watson Bay) and scaled interaction duration (Duration: time spent interacting with client in seconds) were included as main effects. All interactions included in the model are presented. Asterisks depict significant effects ($\alpha = 0.05$).

**Source of variation**	χ^2	**P**
**Main Effects**		
Size	15.21	0.0001 *
Treatment	0.37	0.541
Site	1.43	0.232
Duration	28.73	< 0.0001 *
**Two-way interactions**		
Size X Treatment	4.17	0.041
Size X Site	8.61	0.003 *
Treatment X Site	3.32	0.069
**Three-way interaction**		
Size X Treatment X Site	7.21	0.007 *

Table S3. Mean number of interactions (± SEM) for cleaner filmed at each site (Site), within each site for each treatment (Treatment), and within each treatment for each client size (Client Size).

Site	Treatment	Client Size
High Social complexity: 27.1 ± 2.6	Cortisol: 30.1 ± 3.7	Big: 29.3 ± 6.7<br>Small: 30.9 ± 3.6
	Saline: 24.1 ± 3.7	Big: 20.0 ± 4.0<br>Small: 28.2 ± 6.2
Low Social complexity: 33.4 ± 3.8	Cortisol: 34.2 ± 5.0	Big: 22.8 ± 4.5<br>Small: 45.7 ± 7.4
	Saline: 32.7 ± 5.7	Big: 25.0 ± 9.8<br>Small: 40.3 ± 5.3

Supplemental Methods

Baseline cortisol sample preparation from fish whole bodies:

Cortisol determination was carried out by ultra-high pressure liquid chromatography-tandem mass spectrometry (UHPLC-MS/MS). Samples were injected in an Acquity UPLC™ coupled to a Xevo TQ-S triple quadrupole (Waters, Milford, MA, USA) through an electrospray (ESI) interface. The equipment was controlled by Masslynx™ v4.1. Separation was performed at a flow rate of 0.4 ml/min on an Acquity UPLC BEH C18 column (50x2.1mm i.d., 1.7 µm particle size, Waters) thermostated at 30°C. Mobiles phases consisted of water and formic acid 0.05% (mobile phase A) and acetonitrile and formic acid 0.05% (mobile phase B). The following gradient program was applied: 10-50% B in 6.5 min, 50-100% B in 0.5 min, holding at 100% B for 1.5 min, and reequilibrating at 10% B for 1.5 min. The injection volume was 5 µL. Detection was performed in ESI positive mode using multiple reaction monitoring, in which specific precursor to product transitions are monitored. For cortisol, the transition 363.2/121.1 was monitored and cone collision energy voltages were set to 35 and 25 V, respectively. For cortisol-d4, the transition 367.2/121.1 was monitored and cone and collision voltages were set to 30 and 25 V, respectively. Source parameters were as follows: capillary voltage 1.5 kV, source temperature 150 °C, desolvation gas flow and temperature 1000 L/h and 600°C, respectively. Peaks were automatically integrated using Quanlynx™ and normalized to those of the internal standard. Calibration solutions containing cortisol at 0.1, 1, 20, 100 and 250 ng/mL as well as its corresponding internal standard (cortisol-d4) at concentration identical to that present in fish samples were prepared in MeOH 50%. A linear calibration equation weighted by 1/x was applied. Steps used in sample preparation are outlined below:

1) Extraction:

- Weigh the fish in a 50ml Falcon tube and add 9 volumes of methanol (MeOH, e.g. 9 ml for 1 g).
- Extract with Polytron for 3 min.
- Centrifuge, take 1 mL of solution.
- Evaporate in the Speedvac®.
- Reconstitute in 990 µl 5% MeOH containing 10 µl of the internal standard (cortisol-d4, 80 ng/ml).

2) Purification on SPE cartridges (Biotage ABN 25 mg, 3 mL):

- Condition the cartridge with 1ml MeOH 100%
- Equilibrate the cartridge with 1 ml MeOH 5%
- run samples
- Wash the cartridge with 1ml MeOH 5%
- Wash with 1 ml hexane
- Place a 2.0 mL microcentrifuge tube under the cartridge
- Elute with 1ml ethylacetate (EtOAc).

3) Liquid-liquid extraction:

- Add 0.8ml water+NH3 0.05% (pH about 10) to the tube, shake for 20-30 sec, centrifuge, take supernatant
- Add 0.8 ml EtOAc in the tube containing NH3 and repeat the procedure

4) Vacuum pressure drying step (Speedvac®)

- Evaporate EtOAc at 35 degrees to dryness (approx. 30-45 minutes)

5) Transfer to HPLC vials:

- Re-suspend in 75 µl MeOH 50 %
- Vortex for 20 sec
- Place in an ultrasonic bath for 1 minute
- Vortex for 20 sec
- Centrifuge for 3 min at 13’000 RPM
- Collect supernatant and transfer in HPLC vials fitted with conical inserts.
